# Ameliorating Effect on Glycolipid Metabolism of *Spirulina* Functional Formulation Combination from Traditional Chinese Medicine

**DOI:** 10.1155/2022/3910116

**Published:** 2022-07-13

**Authors:** Zifeng Huang, Chang'e Wang, Jie Chen, Xiaoyu He, Kewen Chen, Xiaoqin Jiang, Chao Zhao, Bin Liu

**Affiliations:** ^1^College of Food Science, Fujian Agriculture and Forestry University, 350002 Fuzhou, China; ^2^Engineering Research Centre of Fujian-Taiwan Special Marine Food Processing and Nutrition, Ministry of Education, 350002 Fuzhou, China; ^3^College of Food Science and Technology, Southwest Minzu University, 610000 Chengdu, China; ^4^College of Marine Sciences, Fujian Agriculture and Forestry University, 350002 Fuzhou, China; ^5^Key Laboratory of Marine Biotechnology of Fujian Province, Institute of Oceanology, Fujian Agriculture and Forestry University, 350002 Fuzhou, China

## Abstract

Insulin resistance is the major factor involved in the pathogenesis of type 2 diabetes. Although the oral drug metformin (MH) is widely used to reduce hyperglycemia, it is associated with adverse effects. Therefore, there is an urgent need to search for safe and natural foods that do not cause adverse effects as alternatives to commercial drugs. In this study, the active substances from *Spirulina platensis*, *Grifola frondosa*, *Panax ginseng*, and chromium-rich yeast were used to obtain *Spirulina* functional formulations (SFFs), and its therapeutic effects on mice with glycolipid metabolism disorder (GLD) were investigated. Results showed that SFFs not only improved glycolipid metabolism and reduced inflammation in mice with GLD but also showed good regenerative effects on the liver, jejunum, and cecum tissues. Moreover, SFFs could inhibit the growth of harmful microbes in the intestine and promote the proliferation of beneficial bacteria, thereby promoting the production of short-chain fatty acids and further regulating GLD. Additionally, SFFs significantly increased the expression of INS, INSR, IRS-1, PI3K, AKT-1, and GLUT-4 genes and significantly decreased that of GSK-3*β* in the INS/PI3K/GLUT-4 signaling pathway. Therefore, the findings of this study suggest that SFFs can be further developed as a new class of therapeutic agents against GLD.

## 1. Introduction

Diabetes is a chronic disease characterized by hyperglycemia resulting from insulin deficiency under the condition of islet cell dysfunction or insulin resistance. By 2017, the number of people with diabetes worldwide is close to 250 million, which is about 9% of the adult population worldwide [1]. Currently, the most commonly used drugs for diabetes treatment are biguanides, which act on HbA1c and thiazolidinediones in the liver. However, the long-term use of biguanides is associated with the onset of serious adverse effects and a huge financial burden [2–4]. Novel hypoglycemic treatment approaches have been established as a result of in-depth and ongoing research, such as the use of molecular techniques to study new metabolic pathways, the development of extremely effective new medications, and innovative prognostic strategies [5]. To date, the most effective way to prevent diabetes is to prevent it in advance, such as eating nutritious foods, exercising, maintaining a positive attitude, and choosing appropriate dietary supplements to prevent high blood glucose levels. Natural products are the simplest, effective, and economical option to prevent type 2 diabetes. Drugs available in the market may cause serious side effects or toxicity [5]. Nowadays, traditional Chinese medicine (TCM) is gradually being accepted by people globally [6]. TCM has shown good results in the treatment of diabetes. A recent study has shown that TCM can improve GLD and insulin resistance in a mouse model of type 2 diabetes [7].

*Spirulina platensis* belongs to the genus Cyanobacteria. With its excellent taste and rich nutritional advantages, its products are among the most popular health supplements in the world. A large number of studies have shown that *Spirulina* and its derivatives exert significant therapeutic effects on oxidative stress-induced diseases [8, 9]. Phycocyanin derived from *Spirulina* can be used as a source of drugs for the prevention of chronic kidney disease and its complications [10]. Moreover, *Spirulina* can promote wound healing and its polysaccharides have been shown to have antioxidant properties [11]. Furthermore, *Spirulina* serves as a functional food additive and a drug for the treatment of type 2 diabetes [12]. The mushroom *Grifola frondosa* is used in TCM to treat chronic diseases such as diabetes, hyperlipidemia, and obesity [13]. The polysaccharide derived from *Panax ginseng* has been shown to have antitumor effects [14, 15]. In addition, ginsenosides have been reported to have hypoglycemic, antioxidant, antifatigue, anticancer activity, and anti-inflammatory effects [16, 17]. Chromium, an important trace element, maintains glucose metabolism; however, excessive consumption of inorganic chromium can easily lead to human poisoning. Chromium toxicity is greatly reduced by converting it into organic chromium. A study showed that yeast is a good carrier of chromium [18]. Chromium-rich yeast is an artificially developed functional yeast that acts as a natural blood glucose regulator and antioxidant.

In TCM, various herbs are often combined into multiherb formulations to enhance efficacy as well as reduce toxicity or adverse effects. Combining *S. platensis* with other herbs may be a better strategy to treat GLD than using *S. platensis* alone. However, to date, few studies have described the role of *S. platensis* functional formulations (SFFs) in lowering blood glucose and lipid levels and in regulating the gut microbiota. SFFs play a key role in regulating glucose and lipid metabolism, restoring gut microbial function, and improving fatty liver symptoms. In this study, *S. platensis* was used as the main material, and *G. frondosa*, *P. ginseng*, and chrome-rich yeast were used as additives. *S. platensis*, *G. frondosa*, and *P. ginseng* were extracted to obtain their respective extracts under different extraction conditions. This study is aimed at assessing whether SFFs have a similar effect to drugs in treating GLD. The findings of this study are expected to provide insights into the beneficial effects of *Spirulina* in the treatment of GLD.

## 2. Materials and Methods

### 2.1. Extraction of Active Compounds

*S*. *platensis* was purchased from Xindaze *Spirulina* Co., Ltd. (Fuzhou, China), and *G. frondosa* and *P. ginseng* were provided by the National Research Center for Mycorrhizal Engineering Technology, Fujian Agricultural and Forestry University. Chromium-enriched yeast (chromium content as 2 mg/g) was purchased from Jiangsu kingheyuan Bioengineering Co., Ltd. The raw materials were mixed with 10 times the volume of 95% ethanol and extracted at 45°C for 1 h with ultrasonic-assisted extraction to obtain 95% ethanol extract of *S*. *platensis* (SE95) and 95% ethanol extract of *G. frondosa* (GE95). The residues were mixed with 10 times the volume of 60% ethanol and were subjected to ultrasonic-assisted extraction for 1 h at 45°C to obtain 60% ethanol extracts of *S*. *platensis* (SE60) and *G. frondosa* (GE60). Next, the residues were mixed with 10 times the volume of deionized water and were subjected to ultrasonic-assisted extraction at 80°C for 1 h to obtain the *S*. *platensis* water extract (SW) and *G. frondosa* water extract (GW). Finally, the residues were mixed with 10 times the volume of ultrapure water, added with 2% compound protease, and hydrolyzed at 37°C for 1 h to obtain the enzymatic hydrolyzed extracts of *S*. *platensis* (SH) and *G. frondosa* (GH). *P. ginseng* water extract (PW) was extracted using the above water extraction method. According to the regulations of the National Health Commission of PRC, the maximum daily intake of humans was converted into that of mice. Then, 2 g of *S*. *platensis*, 2.5 g of *G. frondosa*, and 0.6 g of *P. ginseng* were used for extraction, and the obtained extracts were matched to prepare SFFs. The compound containing *S*. *platensis* extract, *G. frondosa* extract, and chromium-rich yeast was named SGC, and SGCG included *S*. *platensis* extract, *G. frondosa* extract, *P. ginseng* extract, and chromium-rich yeast.

### 2.2. LC-MS Analysis of SFFs

The main chemical compounds in SGC and SGCG were determined by methods described previously [19]. Firstly, we take an appropriate amount of sample in a 2 mL EP tube, add 0.6 mL 2-chlorophenylalanine (4 ppm) methanol (-20°C), and vortex for 30 s. When the sample size was less than or equal to 100 mg, the extraction system was halved. Secondly, we add 100 mg glass beads, put them into the tissue grinder, and grind for 60 s at 55 Hz. Thirdly, we ultrasound for 15 min at room temperature. Finally, we centrifugate at 12000 rpm at 4°C for 10 min, take 300 *μ*L supernatant and filter through 0.22 *μ*m membrane and then add the filtrate into the detection bottle for LC-MS detection.

### 2.3. Animal Model Construction

Fifty 5-week-old SPF Kunming male mice were obtained from Wu's Animal Center (Fuzhou, China). Mice were fed for one week on a 12 h day/night cycle at 25°C and 58% humidity. The mice were fed normal feed (12.5% fat, 23.5% protein) during the acclimatization period in strict accordance with the experimental animal welfare standards and ethical review procedures. After one week of acclimatization, the mice were allowed to fast for 24 h. Ten mice were randomly selected by intraperitoneal injection of physiological saline, and the other 40 mice were injected with streptozotocin (STZ, 45 mg/kg). If fasting blood glucose (FBG) values were greater than 11.1 mmol/L, the animal model of glucose and GLD was considered successfully established. Mice were randomly divided into four groups (model, metformin (MH), SGC, and SGCG) and fed a high-sugar and high-fat diet (HFD, 4% cholesterol, 21% sucrose, 66% normal diet, and 9% lard). Diet, water intake, and body weight were monitored at 0, 2, and 4 weeks.

### 2.4. Serum Collection and Testing

Mice were sacrificed by enucleation of the eyeball after 4 weeks of the experiment. Serum lipid levels were determined according to the kit instructions provided by Wuhan Purity Biology Co., Ltd. In brief, fresh blood was allowed to stand for 25 min, serum samples were obtained by centrifugation, and relevant serum parameters were measured.

### 2.5. Measurement of FBG Levels and Oral Glucose Tolerance Test (OGTT) in Mice

At the beginning of 0, 2, and 4 weeks, FBG values were measured by collecting blood from the tip of each animal's tail after 12 h of fasting. At the end of week 4, the OGTT was performed [20] and the area under the curve (AUC) was calculated [21] afte*r* the mice had undergone fasting for 12 h.

### 2.6. Mouse Liver, Cecum, and Jejunum Sample Collection and Liver Index Assay

After the mice were dissected, their liver, cecum, and jejunum tissues were quickly removed, and the liver tissues were weighed, prefrozen with liquid nitrogen, and stored in a -80°C freezer for further analysis. Then, the liver samples were homogenized and centrifuged. The supernatant was collected to determine liver TC, TG, LDL-c, and HDL-c levels, and the remaining part was stored at -80°C for future use.

### 2.7. Liver, Cecum, and Jejunum Histopathological Observations

Partial samples of the fresh liver, cecum, and jejunum tissues were collected and fixed in a 4% paraformaldehyde solution and embedded in paraffin. They were cut into 4 *μ*M slices, stained with hematoxylin and eosin, and examined for morphological changes under a microscope.

### 2.8. High-Throughput Sequencing Analysis

Total DNA was extracted using the QIAamp DNA Stool Mini Kit (Qiagen, Hilden, Germany), and the V3-V4 region was amplified using the following primer set: forward primer: 5′- CCTACGGRRBGCASCAGKVRVGAAT-3′ and reverse primer 5′- GGACTACNVGGGTWTCTAATCC-3′. The 16S rRNA sequence was obtained after amplification.

### 2.9. Reverse Transcription-Quantitative Polymerase Chain Reaction (RT-qPCR)

Total RNA was extracted from liver samples using a commercial RNA Extraction Kit (Takara, Kusatsu, Japan). Complementary DNA (cDNA) was prepared using the RT Reagent Kit in a PCR system (Proflex™ PCR, Thermo Fisher Scientific, Singapore). Primers for INS, IRS-1, PI3K, AKT-1, GLUT-4, GSK-3*β*, IL-2R, AMPK-*α*, SREBP-1c, FAS, ACC, SCD1, FOXP3, NF-*κ*B, and STAT5 were designed according to the primer designing principles set by NCBI. *β*-Actin was used as a reference, and relative expression values were calculated using the delta delta Ct (2^−*ΔΔ*Ct^) method. All relevant primer sequences are listed in Table S1.

### 2.10. Western Blotting (WB)

Total proteins were extracted from liver samples, and protein concentrations were measured using the BCA Kit (Bio-Rad, Hercules, CA, USA) following the manufacturer's instructions. A sodium dodecyl sulphate-polyacrylamide gel electrophoresis (SDS-PAGE) was prepared, and proteins were transferred to a poly(vinylidene fluoride) (PVDF) membrane (Millipore, USA). Blots were developed and images were obtained using the Genome XPQ Chemiluminescence Imaging System (Synoptics Ltd., UK).

### 2.11. Statistical Analysis

All experimental data were processed in GraphPad Prism (version 7.0) software. All values were expressed as the mean ± standard deviation (SD, *n* = 10). Differences among multiple groups were assessed using one-way analysis of variance (ANOVA) and Tukey's test. Statistical significance was set at *p* < 0.05. The STAMP graphical software package was used to analyze differences in the microbiota among the groups. The correlation between short-chain fatty acids (SCFAs), genes related to glucose and lipid metabolism, and biochemical indices was analyzed by TBtools software.

## 3. Results

### 3.1. Characterization of Potent Major Compounds

The main compounds identified in SGC and SGCG using LC-MS analysis are listed in Table S2 and Table S3. LC-MS analysis has demonstrated the proposed presence of polyunsaturated fatty acids compounds.

### 3.2. Effects of SFFs on Levels of Body Weight, Blood Glucose, and OGTT

**Figure 1:**
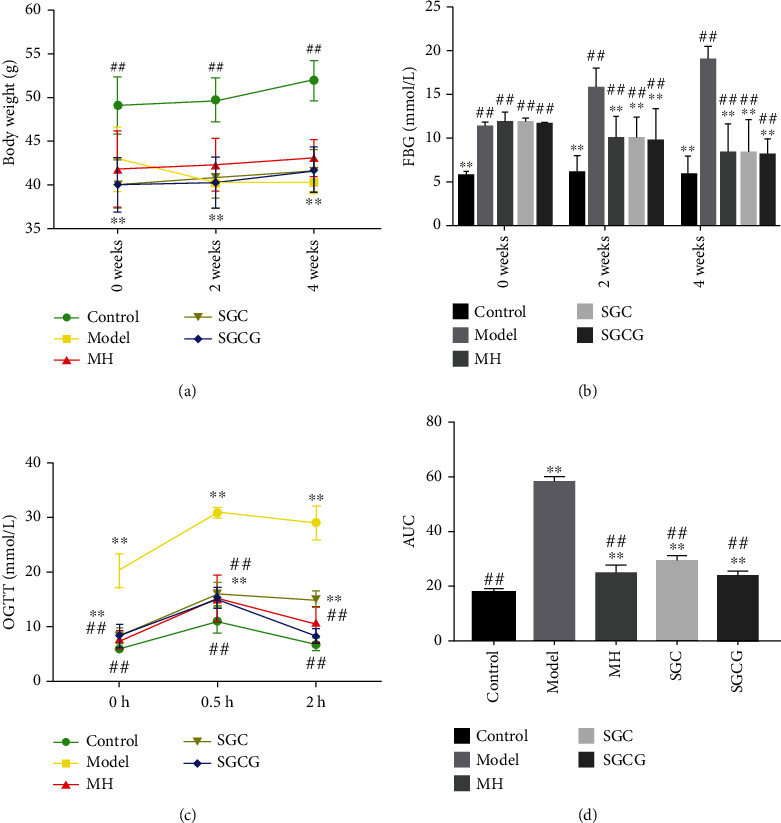
Effects of SFFs on body and serum biochemical indications in different groups. (a) Body weight at 0, 2, and 4 weeks. (b) FBG levels at 0, 2, and 4 weeks. (c) OGTT levels at four weeks. (d) AUC of OGTT at four weeks. Note: the value is the mean ± SD (*n* = 10); compared with the model group, ^##^*p* < 0.01, ^#^*p* < 0.05; compared with the control group,  ^∗∗^*p* < 0.01,  ^∗^*p* < 0.05).

The body weight of mice with GLD showed a downward trend (Figure 1(a)). The results showed that SFFs was effective in inducing weight recovery, which was consistent with our previous conclusion that *Spirulina* alcohol extract could reduce HFD-induced lipid metabolism disorders [22]. Therefore, we deduced that SFFs intake could promote energy metabolism and alleviate lipid metabolism disorders. Compared with those in control mice, FBG levels in GLD mice were significantly increased (*p* < 0.01) (Figure 1(b)**)**. After 2 weeks of SFFs intervention, FBG levels in the SGC and SGCG groups were significantly lower than those in the model group (*p* < 0.01). After four weeks of SFF gavage, FBG levels continued to decline in the SGC and SGCG groups. According to the OGTT index in Figure 1(c), blood glucose levels increased in all mice injected with glucose for 0.5 h. After 2 h of glucose injection, compared with those in the model group, blood glucose levels in the MH, SGC, and SGCG groups decreased significantly, and blood glucose levels in the SGCG group almost recovered to 0 h level. Interestingly, SGCG was more effective than MH in stabilizing blood glucose levels (Figure 1(d)).

### 3.3. Effect of SFFs on Serum Lipid Levels, Insulin Function Index, and Inflammatory Factors

**Figure 2:**
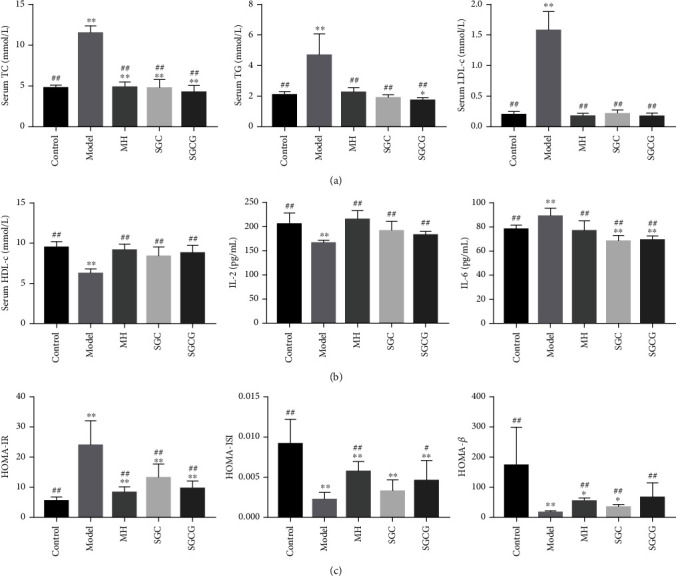
(a) Serum TC, TG, LDL-c, and HDL-c at four weeks in different groups. (b) Inflammatory factors (IL-2 and IL-6). (c) Insulin function index (HOMA-IR, HOMA-ISI, and HOMA-*β*). Note: the value is the mean ± SD (*n* = 10); compared with the model group, ^##^*p* < 0.01, ^#^*p* < 0.05; compared with the control group, ^∗∗^*p* < 0.01, ^∗^*p* < 0.05).

Lipid levels were significantly different between the model and control groups (*p* < 0.01) (Figure 2(a)). The results showed that SFFs could stably increase the level of serum HDL-c (*p* < 0.01) and could effectively reduce the serum TC, TG, and LDL-c levels (*p* < 0.01) compared with the model group. The effect of SGCG on stabilizing blood lipid levels was better than that of SGC (*p* < 0.05). Moreover, serum IL-2 and IL-6 levels were significantly different between the model and control groups (*p* < 0.01). Additionally, serum IL-2 levels in the SGCG group were lower than those in the SGC group. Therefore, SFFs could reduce blood glucose levels and improve inflammatory cytokine levels. The levels of HOMA-*β*, HOMA-IR, and HOMA-ISI in the model group were opposite to those in other groups, indicating the existence of insulin resistance in the model group. After four weeks of intervention, HOMA-*β*, HOMA-IR, and HOMA-ISI levels were recovered to a certain extent (*p* < 0.01). In other words, SFFs could improve insulin sensitivity and insulin resistance.

### 3.4. Effect of SFFs on Liver Lipid Levels

**Figure 3:**
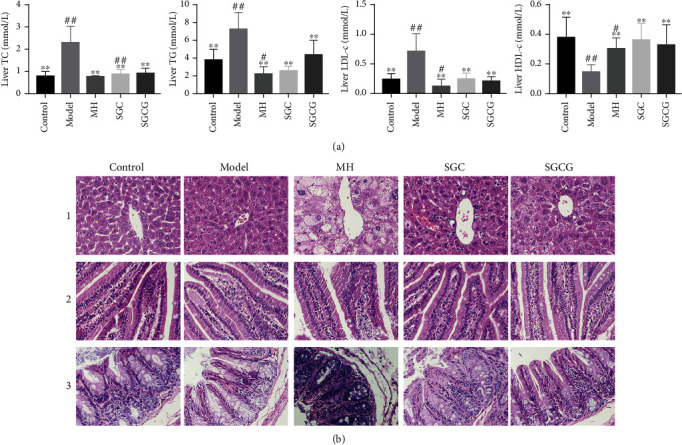
Effects of SFFs on liver and intestine. (a) Liver TC, TG, LDL-c, and HDL-c at 4th week in different groups. (b) Section of liver (1), jejunum (2), and cecum tissue (3). Note: the magnification is 400 times. The value is the mean ± SD (*n* = 10); compared with the model group, ^##^*p* < 0.01, ^#^*p* < 0.05; compared with the control group, ^∗∗^*p* < 0.01, ^∗^*p* < 0.05).

HFD resulted in the excessive accumulation of liver TC, TG and LDL-c and a sharp decrease in liver HDL-c levels in the model group (Figure 3(a)). Excessive intake of HFD increased liver fat accumulation in mice, while SFFs should prevent this accumulation. Compared with the model group, the SGC and SGCG groups showed a significant downward trend in liver lipid levels (*p* < 0.01). In particular, the level of liver HDL-c in the SGC and SGCG groups was significantly increased (*p* < 0.01).

### 3.5. Effect of SFFs on Histomorphology

Histomorphological analysis revealed that hepatocytes of the control group were arranged neatly, with complete margins and clear contours. The hepatic nuclei were clear and radially arranged (Figure 3(b)). Compared with the control group, hepatocyte necrosis and lipid droplet accumulation were obvious in the model group. After SFF gavage, liver injury was significantly decreased, especially in the SGCG group. Compared with the model group, the SCG and SGCG groups exhibited normal structure of hepatocytes with radial shape and a mild degree of hepatic lipemia. These results suggested that SFFs could improve the symptoms of fatty liver in mice to some extent. Compared with that in other groups, the structure of jejunum in the control group showed a complete and orderly villi structure and a thin lipid layer. Compared with other groups, the model group exhibited irregularly arranged gut cells, ruptured gut villi, a considerably thinner gut wall was significantly thinner, and a thickened lipid layer. In addition, the jejunum structure was restored after SFF gavage. Compared with those in the model group, cecal tissue cells in the control group were normal. However, the cecum in the model group had obvious chorionic rupture and membrane ulcer, with significantly damaged tissue. These results suggested that SFFs had a protective effect on these lesions to varying degrees and reduced HFD-induced chronic liver injury by maintaining gut mucosal barrier function.

### 3.6. Effect of SFFs on SCFAs in Cecal Contents

**Figure 4:**
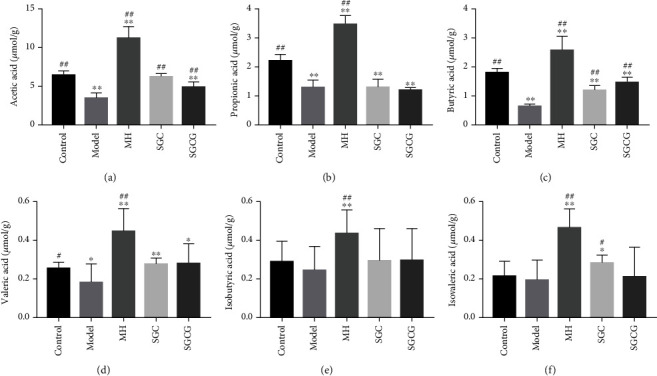
Analysis of SCFAs in mice gut contents: (a) acetic acid, (b) propionic acid, (c) butyric acid, (d) valeric acid, (e) isobutyric acid, and (f) isovaleric acid. Note: the value is the mean ± SD (*n* = 10); compared with the model group, ^##^*p* < 0.01, ^#^*p* < 0.05; compared with the control group, ^∗∗^*p* < 0.01, ^∗^*p* < 0.05).

HFD can cause significant changes in the levels of gut metabolites. The results showed that SFFs could significantly increase the production of SCFAs (Figure 4). Compared with that in the model group, the acetic acid content of the SGCG and SGC groups was increased by 123.60% and 189.54%, respectively (*p* < 0.01). The content of butyric acid was increased by 286.28% in the SGCG group and 213.51% in the SGC group (*p* < 0.01). These data showed that SFFs significantly increased SCFA levels.

### 3.7. Effect of SFFs on Key Genes of Glucose and Lipid Metabolism in GLD Mice

**Figure 5:**
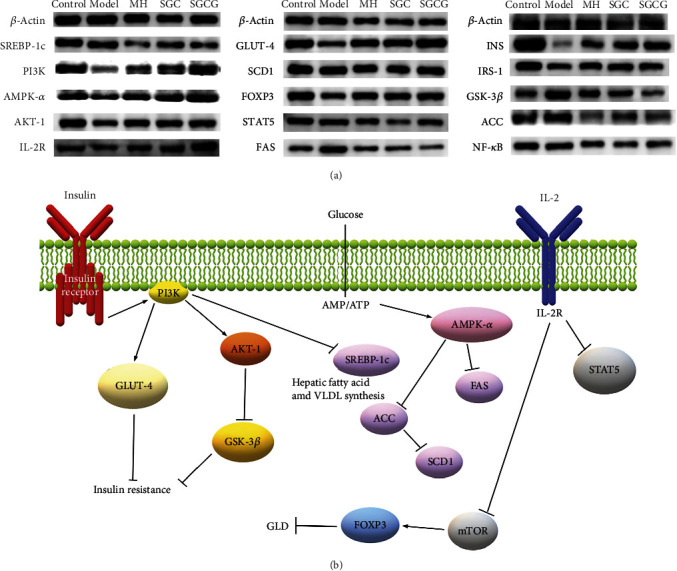
(a) Effects of SFFs on the expression of INS/PI3K/GLUT-4, AMPK-*α*/FAS/SCD, and p^38^MAPK/mTOR/IL-2 signaling pathway-related protein. (b) Schematic representation of the regulatory role of SFFs. Note: arrows indicate facilitation and flat arrows indicate inhibition.

The effects of SFFs on the gene expression of three key proteins in glucose and lipid metabolism pathways, including INS/PI3K/GLUT-4, AMPK-*α*/FAS/SCD, and p38MAPK/mTOR/IL-2, were verified by RT-qPCR assay. The results showed that the expression levels of INSR, IRS-1, PI3K, AKT-1, GLUT-4, IL-2R, FOXP3, and AMPK-*α* in the model group were lower than those in other groups, while the expression levels of *GSK-3β*, SREBP-1c, FAS, ACC, SCD1, mTOR, NF-*κ*B, and STAT5 were higher than those in other groups (Figure 5(a)). These results suggested that HFD could stimulate the internal environment through these three signaling pathways, thereby aggravating liver injury. SFFs could effectively alleviate GLD, and the intervention effect of SCGC was significantly better than SGC. The improvement effect of SFFs on GLD was showed in Figure 5(b). The activation of PI3K could promote the expression of GLUT-4 and then inhibit the insulin resistance. SFFs also promoted the AKT-1 expression, which in turn inhibited GSK-3*β* expression and reduced insulin resistance. And the inhibition of mTOR expression could promote FOXP3 expression and thus alleviate GLD.

### 3.8. Regulation of SFFs on Gut Microbial Structure

All groups showed differences in the gut microbial structure (Figure S1). The distribution of microbial species in the control and MH groups was the same, but it was different from that in the model group. Interestingly, the gut microbial composition in the SGC and SGCG groups was different from that in the other three groups, with the model group showing the most alteration in the gut microbial structure. This suggested that SGC and SGCG could significantly change the gut microbial structure in GLD mice, thus showing a good therapeutic effect.

### 3.9. Analysis of Different Gut Microbial Species in Mice

**Figure 6:**
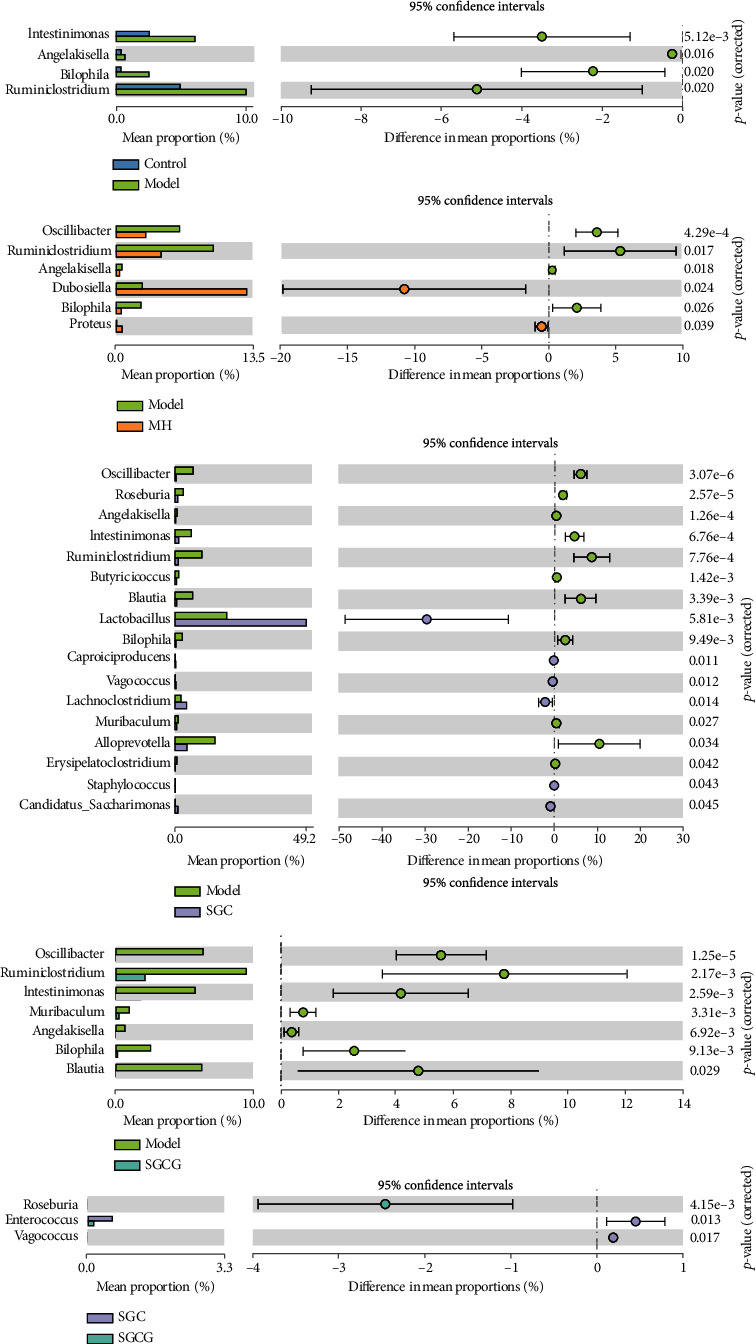
Bar chart of gut content difference and microbiota expansion error of mice in each group.

The microbial species prevalent in the model group were *Intetinimonas*, *Ruminiclostridium*, *Bilophila*, *Angelakisella*, *Osillibacter*, *Angelakisella*, and *Ruminiclostridium* (*p* < 0.05) (Figure 6). After SFF gavage, the relative abundance of *Intestinimonas*, *Ruminiclostridium*, *Bilophila*, *Alloprevotella*, and *Acetatifactor* decreased, while that of *Lactobacillus*, *Bacteroides*, and *Lachnoclostridium* significantly increased (*p* < 0.05). The abundance of the above species was significantly different from that in the model group. Therefore, the composition of gut microbiota in GLD mice changed after SFF gavage.

### 3.10. Regulation of SFFs on Gut Microbiota and Related Biochemical Indices

**Figure 7:**
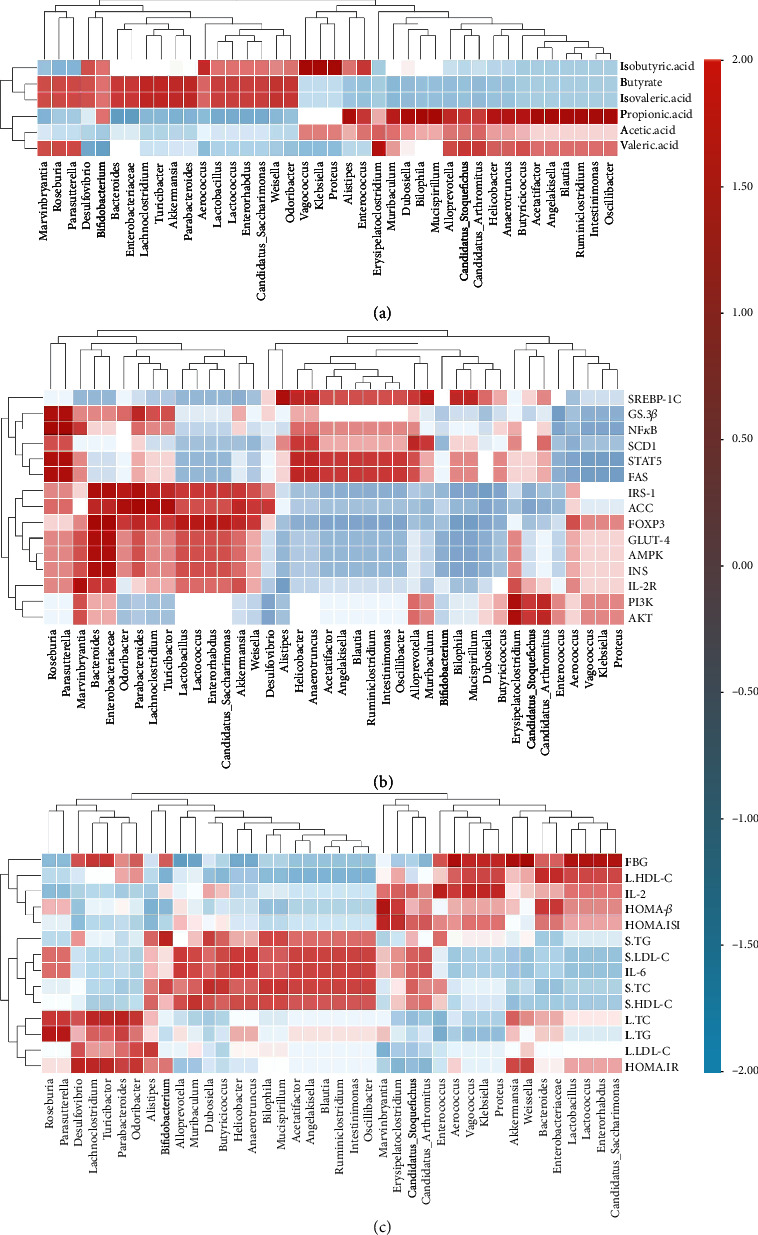
Heatmap comparison and hierarchical clustering dendrogram between bacteria and (a) SCFAs, (b) keys of glucose and lipid metabolism, and (c) key biochemical indices. Note: red represents positive correlation; blue represents negative correlation; the depth of the color indicates the degree of association.

The content of acetic acid, propionic acid, butyric acid, and isovaleric acid was positively correlated with the abundance of *Capriciproducens*, *Parvibacter*, *Bifidobacterium*, *Acetitomaculum*, *Lactococcus*, *Enterrhabdus*, and *Enterococcus* and was negatively correlated with the abundance of *Blatuia*, *Roseburia*, *Paralytterella*, *Muribaculum*, *Helicobacter*, and *Alloprevotella* (Figure 7(a)). The bacteria that were negatively correlated with biochemical indices were negatively correlated with the content of SCFAs. This further proved that SFFs promoted the growth of beneficial microorganisms, inhibited the growth of harmful bacteria, and restored the production of SCFAs *in vivo*, in order to stabilize the intestinal environment and maintain the health of the host. The abundance of *Akkermansia*, *Intestinimonas*, *Enterrhabdus*, *Enterococcus*, and *Parabactoids* was positively correlated with the protein expression levels of PI3K, INSR, AKT-1, GLUT-4, INS, IL-2, IL-2R, FOXP3, mTOR, IRS, and AMPK-*α*. However, the abundance of the above species was negatively correlated with the expression levels of SCD1, FAS, GSK-3*β*, ACC, IL-6, SREBP-1c, and NF-*κ*B (Figure 7(b))). The abundance of these microbial species was significantly reduced after SFF gavage. The abundance of *Ruminiclostridium*, *Intestinimonas*, *Enterrhabdus*, *Bilophila*, *Alloprevotella*, *Helicobacter*, *Parasutterella*, *Oscillibacter*, *Angelakisella*, *Weissella*, and *Candidatus*-*Saccharimonas* was positively correlated with the levels of FBG, IL-6, and HOMA-IRI. Moreover, it was positively correlated with serum levels of TC, TG, and LDL-c and hepatic levels of TC, TG, and LDL-c. In contrast, the abundance was negatively correlated with serum HDL-c, liver HDL-c, serum insulin *β*-cell functional index, and IL-2 levels (Figure 7(c)). After SFF gavage, the abundance of the above microbial species was significantly reduced. This result proved that gut microbial abundance was closely related to the levels of biochemical indices of glucose and lipid metabolism.

## 4. Discussion

Existing studies have shown that *S. platensis*, *G. frondosa*, and *P. ginseng* exert some therapeutic effects on GLD. However, few studies have suggested the combined use of these three substances in the treatment of glucose and lipid metabolism disorders. Therefore, in this study, we explored whether adding PW to *S. platensis*, *G. frondosa*, and chromium-rich yeast can lead to a better therapeutic effect. Results showed that SGCG had a better therapeutic effect on GLD mice because the weight of GLD mice showed a decreasing trend. After four weeks of SFF gavage, the symptoms of GLD in mice were significantly improved. According to the results of serum lipid profiling, SFFs could restore the biochemical indices of serum lipid metabolism to normal levels in GLD mice. It is well known that TC, TG, LDL-c, and HDL-c are closely related to metabolic syndrome [23, 24]. According to the results of blood glucose measurement, the hypoglycemic effect of SFFs was more obvious than that of *S. platensis*. Excessive TG in the blood is transported to the liver, leading to liver fat accumulation and an increased burden of liver diseases [25]. SFFs could balance glucose and lipid metabolism and restore the HFD-induced fatty liver.

Abnormal levels of key biochemical indices may lead to morphological changes in mice. The model group exhibited significant structural changes of the jejunum and cecum, separated glands and villi, broken and loosely arranged villi, a thicker fat layer, and the presence of enteritis. These symptoms could be effectively reversed after SFF gavage, indicating that SFFs could not only effectively repair liver tissue damage and inhibit the occurrence of fatty liver but also significantly improve the physiological structure of the cecum and jejunum. These results suggest the anti-inflammatory role of SFFs in tissues.

The gut microbiota constitutes the complex network of microorganisms in the gut which are closely related to human physiology [26]. Some key gut microbial species associated with GLD, hepatic lipid accumulation, and insulin resistance have been identified as biomarkers of GLD [27]. Furthermore, gut probiotics not only improve insulin resistance but also reduce obesity by interacting with intestinal epithelial cells [28]. This study showed that SFFs could restructure the gut microbiota and reduce gut injury in mice, thus improving GLD symptoms and facilitating the microbiota to exert beneficial effects on the host. The species richness of the control group was higher than that of the SFF groups. However, the species richness of the model group was low, indicating that SFFs enriched the gut microbial diversity of GLD mice. The species diversity of the SGC and SGCG groups was higher than that of the model group. The control and MH groups were located between the model and SGC groups (Figure S1). The microbiota structure of the model group was significantly different from that of the control group, indicating that long-term HFD had adverse effects on the microbiota structure of mice. Studies showed that 95% ethanol extract of *Spirulina* can enrich *Alloprevotella* and *Ruminococcus* and also reduce the abundance of *Firmicutes* and improve the abundance of *Bacteroidetes* [19]. 95% ethanol extract of *G. frondosa* can increased the relative abundance of *Butyricimonas* [29]. *G. frondosa* water extract can increased the relative abundance of *Akkermansia*, *Lactobacillus*, and *Turicibavter* [30]. SFF intake could improve and maintain the microbiota structure to a certain extent. SFFs increased the abundance of beneficial microbiota in the gut of GLD mice, such as *Bacteroides*, *Akkermansia*, *Alloprevotella*, *Bifidobacterium*, and *Parapprevotella*, indicating that SFFs regulated and improved their microbiota structure, which was in accordance with the results of a previous study [31]. It also indicated that SFFs can enhance the function of a single substance. The increase of probiotics increased the content of SCFAs. After SFF gavage, the content of SCFAs, such as acetic, propionic, butyric, isobutyric, and isovaleric acids, was significantly increased in SGC and SGCG groups (*p* < 0.01), indicating that SFFs may regulate the content of SCFAs by increasing the abundance of beneficial bacteria. To sum up, SFFs might play a key role in regulating glucose and lipid metabolism, restoring gut microbial function, and improving fatty liver symptoms.

In terms of glucose metabolism, compared with the model group, SFFs significantly increased the mRNA transcription levels of INS, INSR, IRS-1, PI3K, AKT-1, and GLUT-4 and significantly decreased the mRNA transcription level of GSK-3*β* (*p* < 0.01). When INS binds to INSR on the cell membrane, it promotes the phosphorylation of INSR [32]. Downregulation of INSR expression can trigger insulin resistance [33]. In addition, GLUT-4 is the terminal signal of intracellular insulin signal transduction [34, 35]. SFFs could activate the INS signal transduction pathway and alleviate insulin resistance. GSK-3*β* is the most important exogenous activator that can improve insulin sensitivity as well as inhibit liver inflammation and lipid deposition [36]. SFFs could reduce blood glucose levels and repair liver damage, which may be related to the regulation of GSK-3*β* expression. In terms of lipid metabolism, compared with the model group, SFFs significantly increased the mRNA expression level of AMPK-*α* and significantly decreased the mRNA expression levels of SREBP-1c, FAS, ACC, and SCD1 (*p* < 0.01). AMPK-*α* is an important target for therapeutic intervention of GLD; AMPK-*α* and its downstream regulatory factors such as SREBP-1c, FAS, ACC, and SCD1 regulate glucose and lipid metabolism [37, 38]. ACC, a key enzyme related to lipid metabolism, determines the synthesis rate of fatty acids. SCD1 is a new target for therapeutic intervention of steatohepatitis and inflammation [39]. After SFF gavage, the expression levels of AMPK-*α* and ACC were similar to those in the control group. SFFs primarily inhibit hepatic glucose production by activating AMPK-*α*. In addition, the expression of SREBP-1c is significantly correlated with altered TG levels, thereby inhibiting the synthesis of cholesterol and fatty acids [40]. In our study, the activation of liver SREBP-1c promoted the accumulation of TC and TG. The expression levels of SREBP-1c, FAS, and ACC in the SGC and SGCG groups were lower than those in the control group. In terms of inflammatory regulation, SFFs significantly increased the mRNA expression levels of IL-2, IL-2R, and FOXP3 and significantly decreased the mRNA expression levels of NF-*κ*B, STAT5, and IL-6 (*p* < 0.01). Studies have shown that IL-2, as an upstream regulator of STAT5, can regulate STAT5 expression [41]. The levels of FBG, TC, TG, and other indicators were significantly decreased, indicating that the decreased IL-2 expression may be related to hyperglycemia and insulin resistance. This result also confirmed that SFFs could inhibit STAT5 expression by upregulating IL-2 expression. IL-2 expression could interfere with the mTOR signaling pathway, thus activating FOXP3 expression and reducing the inflammatory response. These results suggested that SFFs could induce anti-inflammatory effects by regulating the p38MAPK/mTOR/IL-2 signaling pathway. The expression of NF-*κ*B and its inducer IL-6 was significantly downregulated after SFF gavage. SFFs regulated the expression of NF-*κ*B and its downstream target IL-6, thereby reducing inflammation [42]. The expression levels of INS, AMPK-*α*, and their downstream were measured by western blotting. The results showed that the protein expression level was consistent with the mRNA transcription level. SFFs may improve GLD by regulating INS/PI3K/GLUT-4, AMPK-*α*/FAS/SCD, and p38MAPK/mTOR/IL-2 pathways [43], thus showing strong anti-GLD activity.

The study showed that HFD led to a significant decrease in the abundance of beneficial bacteria in the gut and a significant increase in the abundance of harmful bacteria. This inhibited the gut's ability to produce acid, promoted the absorption of glucose and lipids, and led to metabolic disorders. SFFs could activate AMPK-*α*, which is involved in autophagy and inflammation, affect the gut environment, and negatively regulate glucose and lipid metabolism. Furthermore, this study demonstrated for the first time the effect of SFFs on insulin, energy metabolism, and inflammatory metabolism pathways, as well as the molecular mechanism by which SFFs modulate the glucose and lipid metabolism. SFFs could stimulate insulin secretion, activate AMPK-*α*, promote glucose transport, and inhibit insulin resistance, gluconeogenesis, and hepatic adipose accumulation. Therefore, SFFs could act on multiple targets to enhance the regulation of GLD.

## 5. Conclusions

SFFs could effectively improve serum and liver lipid levels and improve the symptoms of enteritis in GLD mice. SFF gavage could not only improve the abundance of gut beneficial microbiota but also regulate and improve the gut microbiota structure of GLD mice. At the same time, SFFs regulated the mRNA and protein expression of key genes involved in metabolic pathways through multiple targets and inhibited abnormal glucose and lipid metabolism and inflammatory responses. The mRNA levels of IRS-1, PI3K, GLUT-4, and AKT-1 were increased and those of GSK-3*β* were decreased, indicating that SFFs has the potential to improve GLD. Therefore, our findings indicated that SFFs is a promising food supplement to be added to the hypoglycemic functional food. This study only proved the hypoglycemic ability of SFFs, and further research should be devoted to the specific hypoglycemic mechanism of SFFs.

## Data Availability

The datasets generated for this study are available on request to the corresponding authors.

## Abbreviations

MH: Metformin
SFFs: Spirulina functional formulations
GLD: Glycolipid metabolism disorder
TCM: Traditional Chinese medicine
SE95: 95% ethanol extract of *S. platensis*
SE60: 60% ethanol extract of *S. platensis*
SW: *S. platensis* water extract
SH: The enzymatic hydrolyzed extracts of *S. platensis*
GE95: 95% ethanol extract of *G. frondosa*
GE60: 60% ethanol extract of *G. frondosa*
GW: *G. frondosa* water extract
GH: The enzymatic hydrolyzed extracts of *G. frondosa*
PW: *P. ginseng* water extract
STZ: Streptozotocin
FBG: Fasting blood glucose
HFD: High-sugar and high-fat diet
OGTT: Oral glucose tolerance test
AUC: The area under the curve
SCFAs: Short-chain fatty acids.

## Supplementary Materials

Supplementary Materials Figure S1: the differences in the gut microbial structure among each group of mice. Table S1: all relevant primer sequences are listed. Table S2: the representative MS fragments of major compounds of SGC. Table S3: the representative MS fragments of major compounds of SGCG.
