# Supplementary material for: Ameliorating Effect on Glycolipid Metabolism of Spirulina Functional Formulation Combination from Traditional Chinese Medicine
Source: Oxid Med Cell Longev. 2022 Jul 13;2022:3910116. doi: 10.1155/2022/3910116 (PMC9300286; doi:10.1155/2022/3910116)
Supplement: Supplementary Materials — Figure S1: the differences in the gut microbial structure among each group of mice. Table S1: all relevant primer sequences are listed. Table S2: the representative MS fragments of major compounds of SGC. Table S3: the representative MS fragments of major compounds of SGCG. [file 3910116.f1.zip › Table S1 Primer sequences (1).pdf]

Table S1 Primer sequences.

Primer name	Forward primer sequence	Reverse primer sequence
ACC	5'-GAATCTCCTGGTGACAATGCTTATT-3'	5'-GGTCTTGCTGAGTTGGGTTAGCT-3'
SCD1	5'-GGGTGCCGTGGGCGA-3'	5'-ACTCAGAAGCCCAAAGCTCAG-3'
FOXP3	5'-CCCCTCTAGCAGTCCACTTCA-3'	5'-AAGTTGCCGGGAGAGCTGAAT-3'
NF-κB	5'-GAGCAACCAAAACAGAGGGG-3'	5'-GGGTAGGATTTCTTGTTCTTCTCAC-3'
STAT5	5'-CACTCCTGTACTTGGTTCGTCA-3'	5'-CCAGGTCAAACTCGCCATCT-3'
AMPK-α	5'-AAGCCGACCCAATGACATCA-3'	5'-CTTCCTTCGTACACGCAAAT-3'
IL-2R	5'-GTTGCACAAAGCCCAAGGTG-3'	5'-AAGGCGAGCCCACTTCCTTAC-3'
GLUT-4	5'-CCTGCCCGAAAGAGTCTAAAGC-3'	5'-ACTAAGAGCACCGAGACCAACG-3'
GSK-3β	5'-TGCGGGAGAACTTAATGCTG-3'	5'-TGCTGCCATCTTTATCTCTGCT-3'
β-actin	5'-TGTCCACCTTCCAGCAGATGT-3'	5'-AGCTCATAACAGTCCGCCTAGA-3'
INS	5'-CTCCTGCTTGAGAACGTGGT-3'	5'-GTGCTCTGCTCACACTTCCT-3'
IRS-1	5'-TAGGCAGCAATGAGGGCAAC-3'	5'-GGTCATTTAGGTCTTCATTCTGCT-3'
PI3K	5'-AGACACCATTACAAAGAAAGCCG-3'	5'-TTTCATTCACTTCTTCCCTTGAGA-3'
AKT-1	5'-ACTCATTCCAGACCCACGAC-3'	5'-CCGGTACACCACGTTCTTCT-3'
SREBP-1c	5'-ACTTTTCCTTAACGTGGGCCT-3'	5'-TGAGCTGGAGCATGTCTTCG-3'
FAS	5'-GTGACCGCCATCTATATCG -3'	5'-TGCTGTCGTCTGTAGTCT-3'
