# Supplementary material for: Ameliorating Effect on Glycolipid Metabolism of Spirulina Functional Formulation Combination from Traditional Chinese Medicine
Source: Oxid Med Cell Longev. 2022 Jul 13;2022:3910116. doi: 10.1155/2022/3910116 (PMC9300286; doi:10.1155/2022/3910116)
Supplement: Supplementary Materials — Figure S1: the differences in the gut microbial structure among each group of mice. Table S1: all relevant primer sequences are listed. Table S2: the representative MS fragments of major compounds of SGC. Table S3: the representative MS fragments of major compounds of SGCG. [file 3910116.f1.zip › Table S2 Representative MS fragments of major compounds of SGC.pdf]

Table S2 Representative MS fragments of major compounds of SGC.

Name	mz	Formula	KEGG	Type	Class
gamma-Aminobutyric acid	104.1079	$C_4H_9NO_2$	C00334	[M+H]+	Carboxylic acids and derivatives
Adenosine	268.1039	$C_{10}H_{13}N_5O_4$	C00212	[M+H]+	Purine nucleosides
Phytosphingosine	318.2994	$C_{18}H_{39}NO_3$	C12144	[M+H]+	Organonitrogen compounds
Glycerophosphocholine	258.1109	$C_8H_{21}NO_6P$	C00670	[M]+	Glycerophospholipids
Alanyl-Tyrosine	253.1180	$C_{12}H_{16}N_2O_4$	NULL	[M+H]+	Carboxylic acids and derivatives
L-Phenylalanine	164.0705	$C_9H_{11}NO_2$	C00079	[M-H]-	NULL
Niacinamide	123.0559	$C_6H_6N_2O$	C00153	[M+H]+	Pyridines and derivatives
L-Threonine	120.0658	$C_4H_9NO_3$	C00188	[M+H]+	Carboxylic acids and derivatives
L-Lysine	147.0771	$C_6H_{14}N_2O_2$	C00047	[M+H]+	Carboxylic acids and derivatives
Pyrrole-2-carboxylic acid	111.0206	$C_5H_5NO_2$	C05942	[M]+	Pyrroles
