# Supplementary material for: Ameliorating Effect on Glycolipid Metabolism of Spirulina Functional Formulation Combination from Traditional Chinese Medicine
Source: Oxid Med Cell Longev. 2022 Jul 13;2022:3910116. doi: 10.1155/2022/3910116 (PMC9300286; doi:10.1155/2022/3910116)
Supplement: Supplementary Materials — Figure S1: the differences in the gut microbial structure among each group of mice. Table S1: all relevant primer sequences are listed. Table S2: the representative MS fragments of major compounds of SGC. Table S3: the representative MS fragments of major compounds of SGCG. [file 3910116.f1.zip › Table S3 Representative MS fragments of major compounds of SGCG.pdf]

Table S3 Representative MS fragments of major compounds of SGCG.

Name	mz	Formula	KEGG	Type	Class
L-Isoleucine	132.1015	$C_6H_{13}NO_2$	C00407	[M+H]+	Carboxylic acids and derivatives
8-O-Acetylharpagide	387.1223	$C_{17}H_{26}O_{11}$	NULL	[M-H2O-H]-	Terpenoids
L-Glutamic acid	148.0600	$C_5H_9NO_4$	C00025	[M+H]+	Carboxylic acids and derivatives
L-Malic acid	133.0130	$C_4H_6O_5$	C00149	[M-H]-	Hydroxy acids and derivatives
Niacinamide	123.0555	$C_6H_6N_2O$	C00153	[M+H]+	Pyridines and derivatives
Piperidine	86.0964	$C_5H_{11}N$	C01746	[M+H]+	Piperidines
3-Hydroxymethylglutaric acid	145.0475	$C_6H_{10}O_5$	C03761	[M+H-H2O]+	Fatty Acyls
L-Tyrosine	182.0825	$C_9H_{11}NO_3$	C00082	[M+H]+	Carboxylic acids and derivatives
Sarcosine	90.0558	$C_3H_7NO_2$	C00213	[M+H]+	Carboxylic acids and derivatives
Aniline	94.0458	C_6H_7N	C00292	[M+H]+	Benzene and substituted derivatives
