# Supplementary figures and images for: Ameliorating Effect on Glycolipid Metabolism of Spirulina Functional Formulation Combination from Traditional Chinese Medicine
Source: Oxid Med Cell Longev. 2022 Jul 13;2022:3910116. doi: 10.1155/2022/3910116 (PMC9300286; doi:10.1155/2022/3910116)

# PCA Analysis

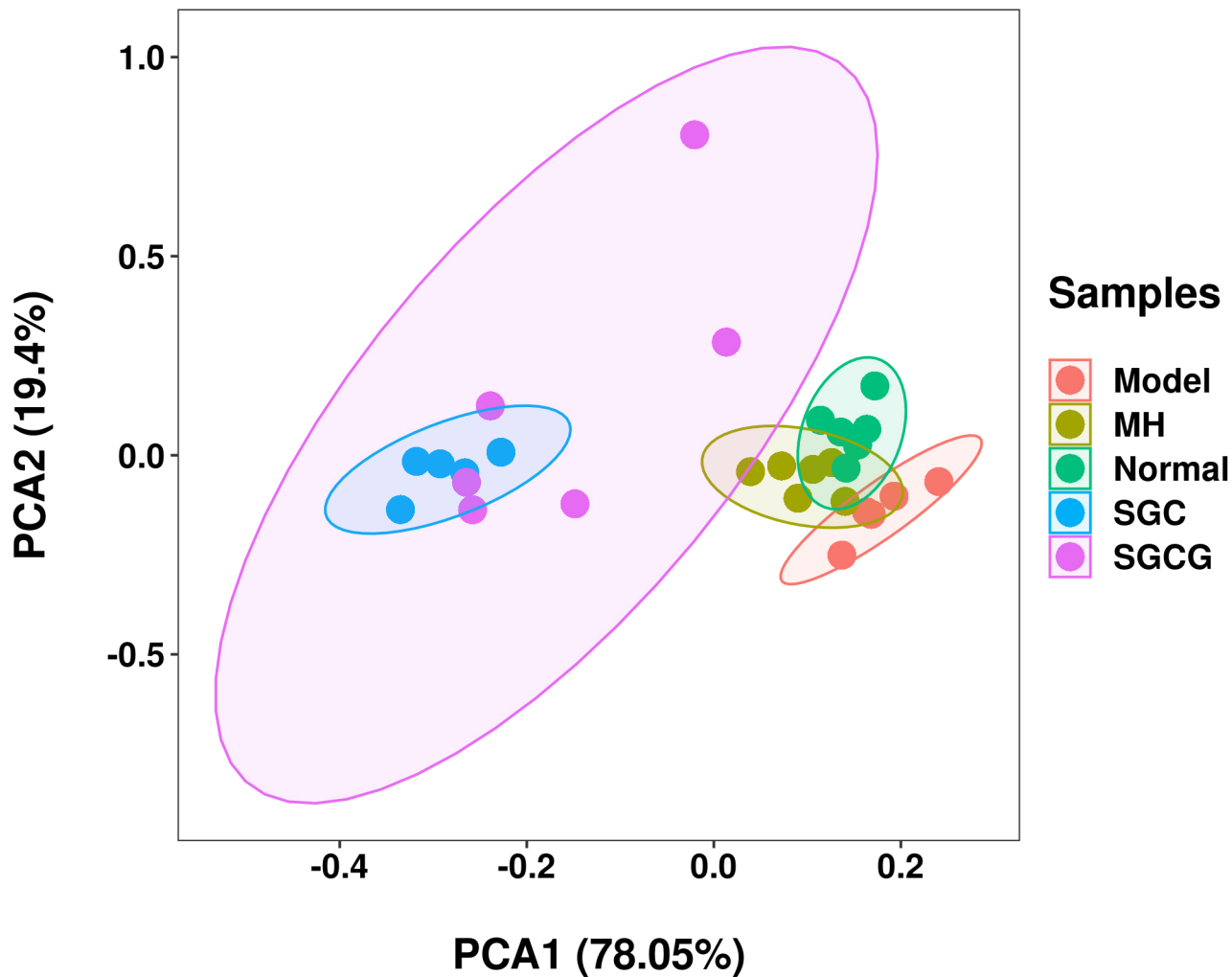

Supplement: Supplementary Materials — Figure S1: the differences in the gut microbial structure among each group of mice. Table S1: all relevant primer sequences are listed. Table S2: the representative MS fragments of major compounds of SGC. Table S3: the representative MS fragments of major compounds of SGCG. [file 3910116.f1.zip › Figure S1 PCA analysis of gut contents flora .pdf]
